# SCAFFOLDING PARENTS TO ACCEPT ADULT CHILDREN’S INTERVENTION

**DOI:** 10.1093/geroni/igz038.1044

**Published:** 2019-11-08

**Authors:** Noriko Toyokawa, Nancy Darling, Teru Toyokawa

**Affiliations:** 1 California State University San Marcos, San Marcos, California, United States; 2 Oberlin College, Oberlin, Ohio, United States

## Abstract

Guided by social-cognitive domain specific theory (Smetana, 1997), this study explored the issue of role reversal in the aging parent-adult child relation when parents are experiencing age-related functional limitations. Data was collected from adult children (N=16, Mage=53.06, SD=6.08) with a living parent of 70 years old or older who participated in a focus group and were analyzed by a directed analysis (Potter & Levine-Donnerstein, 1999). Participants legitimated their intervention into parents’ autonomy when they perceived a potential risk of parents’ health and safety and involvement of those and of others. Eight types of intervention emerged: (1) monitoring and talking with potential risk with parent (2) convincing parents under the name of super power or an authority figure (3) scaffolding parent’s task by teaching skills, (4) scaffolding by sharing role, (5) scaffolding by optimizing environment, (5) overriding parents’ autonomy behind parents, (6) forcefully overriding, (7) giving up parents’ behavioral modification by accepting parents’ lifestyle, and (8) giving up because of discomfort of talking about the issue (i.e., potential risks of parent’s sexual intercourse, parents’ death preparation). Thus, adult children changed their strategies of intervention from monitoring their parents’ behaviors to overriding parents’ autonomy, depending on their appraisal of potential harms of parents’ prudential and moral domains of life and of their own work/family conditions from monitoring to overriding. Adult children’s possible ways of scaffolding in helping their parents accept their children’s interventions as letting parents maintain their psychological autonomy, including communication skills to discuss uncomfortable topics is discussed.

